# Localisation of oestrogen receptors in stem cells and in stem cell‐derived neurons of the mouse

**DOI:** 10.1111/jne.13220

**Published:** 2022-12-12

**Authors:** DeAsia Davis, Ruby Vajaria, Evangelos Delivopoulos, Nandini Vasudevan

**Affiliations:** ^1^ School of Biological Sciences University of Reading Reading UK

**Keywords:** neuronal differentiation, oestrogen receptors, stem cells, subcellular localisation

## Abstract

Oestrogen receptors (ER) transduce the effects of the endogenous ligand, 17β‐estradiol in cells to regulate a number of important processes such as reproduction, neuroprotection, learning and memory and anxiety. The ERα or ERβ are classical intracellular nuclear hormone receptors while some of their variants or novel proteins such as the G‐protein coupled receptor (GPCR), GPER1/GPR30 are reported to localise in intracellular as well as plasma membrane locations. Although the brain is an important target for oestrogen with oestrogen receptors expressed differentially in various nuclei, subcellular organisation and crosstalk between these receptors is under‐explored. Using an adapted protocol that is rapid, we first generated neurons from mouse embryonic stem cells. Our immunocytochemistry approach shows that the full length ERα (ERα‐66) and for the first time, that an ERα variant, ERα‐36, as well as GPER1 is present in embryonic stem cells. In addition, these receptors typically decrease their nuclear localisation as neuronal maturation proceeds. Finally, although these ERs are present in many subcellular compartments such as the nucleus and plasma membrane, we show that they are specifically not colocalised with each other, suggesting that they initiate distinct signalling pathways.

## Graphical abstract

This study explores the localisation of oestrogen receptors (ERs) within organelles in two different cell types. In mES cells, there is low membrane localisation of ERs and high nuclear localisation. All receptors are present in the endoplasmic reticulum and the Golgi apparatus to a similar extent. Upon differentiation to neuronal cells, ERs (particularly the ERα variant, ERα‐36) increase localisation at the plasma membrane and at the endoplasmic reticulum. Regardless of cell type, ERs are not colocalised with each other in any organelle or cell compartment and appear to be separately distributed.

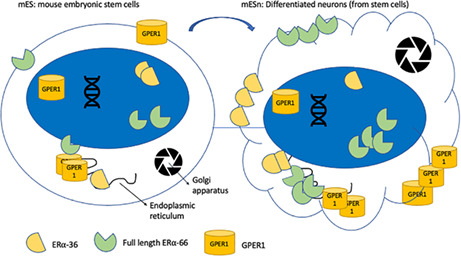

## 1 INTRODUCTION

Oestrogen (17β‐E; E2) plays an important role in reproduction, neuroprotection, vascular and cognitive function^1^, ^2^, ^3^, ^4^ and in the maintenance of sexually dimorphic behaviours by both genomic and nongenomic signalling.^5^ Genomic signalling is a slow signalling mechanism by which oestrogen on binding the nuclear oestrogen receptors ERα and ERβ regulates transcription from target genes.^6^, ^7^ Oestrogen also initiates rapid, nonclassical signalling that involves the activation of multiple protein kinase cascades from plasma‐ membrane localised oestrogen receptors (mERs), particularly in endothelial cells,^8^ breast cancer cells^9^, ^10^ and neurons.^11^, ^12^ In the central nervous system (CNS), rapid signalling by mERs in the nuclei of the social behaviour network regulates sex‐typical social behaviours such as aggression and copulation whereas signalling in limbic nuclei facilitates hippocampus‐dependent learning and memory formation.^13^, ^14^, ^15^

Although the common isoform of Erα, that is, full length ERα‐66 and ERβ were thought initially to be intracellular receptors, a number of studies have shown localisation of these receptors at the plasma membrane, with functional implications.^13^ For example, in astrocytes, the arcuate nucleus and in striatal and hippocampal neurons, ERα can couple to different metabotropic glutamate receptors (mGluR) that is a result of ERα anchoring at the plasma membrane to specific caveolin (CAV) subtypes (reviewed in^16^). Silencing of CAV1^17^ or blockade of mGluR1^18^ in the arcuate nucleus leads to lower localisation of ERα at the plasma membrane, decreases μ‐opioid receptor (MOR) internalisation in the medial preoptic area and lowers lordosis, a measure of female sexual receptivity, in response to E2. Similarly, in astrocytes, the rapid increase in calcium required for the neuroprogesterone release that is important for reproduction is synergistically increased with a combination of mGluR1 agonists and 17β‐E^19^ as well as by ERα‐selective agonists such as PPT (1,3,5‐tris (4‐hydroxyphenyl)‐4‐propyl‐1H‐pyrazole).^20^ This suggests that ERα is anchored at plasma membrane and can functionally act as a mER that initiates rapid nongenomic signalling in the CNS. Consistent with this, a ERα522 mutant incapable of being palmitoylated loses membrane localisation and rapid activation of the pCREB pathway upon 17β‐E treatment^21^ in hippocampal neurons. A variant of ERα called ERα‐36 that lacks the AF‐1 *N*‐terminal domain is a cytoplasmic/plasma‐membrane localised ER, detected in endothelial cells as well as in ERα‐66 positive and ERα‐66‐negative breast cancer cell lines.^22^, ^23^ In triple negative (ERα‐, PR‐, HER2‐) breast cancer cell lines, ERα‐36 promotes proliferation via rapid epidermal growth factor receptor (EGFR)‐extracellular signal‐regulated kinase (ERK) signalling.^22^ In Hec1A endometrial cancer cells, tamoxifen, a first‐line therapy for breast cancer and a mixed agonist/antagonist for ERα‐66 could activate PI3K/PKB signalling in a ERα‐36 dependent manner.^24^ Yet another mER is the G‐protein coupled receptor, GPER1/GPR30, localised at the plasma membrane of SKBR3 cells, which activates EGFR‐ERK signalling via the release of the heparin‐bound EGF^25^; in COS‐7 cells, this interaction with EGFR by GPER1 localised to the endoplasmic reticulum increases calcium flux.^26^ In the rat CA1, GPER1 at the cell membrane of the dendritic spine is anchored to PSD‐95^27^ or to SAP97^28^ and increases social and spatial cognition in mice by rapidly signalling in the hippocampus.^29^ These studies show that apart from the classical nuclear localisation of ERα that is related to its transcriptional role,^11^, ^12^, ^30^ ERs have also been investigated for their presence on the plasma membrane as a prerequisite for membrane‐initiated rapid signalling. Therefore, subcellular localisation is relevant to the type of signalling pathway utilised by these receptors, that is rapid nongenomic signalling in contrast to slower regulation of cellular and behavioural phenotypes.

Investigating the subcellular colocalisation of these receptors also allows us to understand crosstalk between these receptors.^31^ For example, G‐1, a selective GPER1 agonist, deactivates MOR internalisation in the medial preoptic area, signalling in tandem with ERα, in the arcuate nucleus of the hypothalamus (ARH), to facilitate lordosis in female rats.^32^ In non‐neuronal cells, the ability of G‐1 to rapidly increase *c‐fos* expression within an hour in the mouse spermatogonial cell line GC‐1 is dependent on ERα, suggesting that these receptors may signal via in the same pathway.^33^ Recently, ERα‐36 has been shown to physically interact with GPER1 in SKBR3 (ERα‐66 negative) and MCF‐7 breast (ERα‐66 positive) cancer cell lines to inhibit proliferation. This novel GPER1‐ERα‐36 interaction in the cytoplasm is required for the oestrogen inhibition of lipopolysaccharide‐induced inflammation via the inhibition of NF‐κB, suggesting that these receptors can interact with each other in a complex with a cytokine.^34^

Although GPER1, a putative mER, has been localised to the plasma membrane of the dendritic spine,^28^, ^35^ endoplasmic reticulum^28^ and Golgi apparatus^36^, ^37^ in hippocampal and hypothalamic neurons, subcellular localisation of endogenous ERα‐36 has been studied mostly in cancer cell lines^38^ with no report in the CNS. Moreover, subcellular colocalisation of these putative mERs, with the classical receptor ERα has not been studied, although signalling from these receptors may antagonise or synergise with each other to govern final cellular output to oestrogen stimulation.^31^, ^34^ In part, this is due to the difficulty and cost of maintaining primary neuronal cultures from embryonic or neonatal rodent sources.

Neural differentiation protocols predominantly rely on growth factors to induce neural lineage specification. The main morphogens usually employed for motor neuron differentiation are retinoic acid (RA), sonic hedgehog (SHH) and its agonists: smoothened agonist (SAG) and purmorphamine. In most protocols, neural precursor cells (NPC) are first derived from pluripotent stem cells in a process called neural induction. NPC are then patterned into a desired neural lineage with the use of RA and SHH at specific concentrations, followed by the addition of neurotrophic factors which ensures neuronal maturation. The entire process to generate β‐tubulin positive neurons requires 20 days or more.^39^ In this study, we committed mouse embryonic stem cells to neural lineages, by fine tuning a mass suspension protocol by Wichterle and Peljto^40^ to generate embryoid bodies (EBs) within 5 days. This fast and efficient adaptation has produced both astrocytes^41^ and neurons.^42^, ^43^ Hence, the objectives of this study were to examine the subcellular localisation and colocalisation of endogenously expressed ERα, GPER‐1 and ERα‐36 in neurons differentiated from pluripotent mouse stem cells.

## 2 MATERIALS AND METHODS

### 2.1 Cell culture

The male mouse embryonic stem cell line (mES) CGR8 (from inner cell mass of Day 3 mouse embryo, strain 129) was obtained from Sigma Aldrich UK. mES were plated between passage 7 and 13 at 10^6^ cells in gelatine‐coated (0.1%) tissue culture flasks (25 cm^2^) and maintained in media composed of Dulbecco's modified Eagle medium (DMEM) (Gibco, UK), 10% fetal bovine serum (FBS) (Biosera, UK), 1% penicillin/streptomycin (P/S) (Gibco, UK), 1% *L*‐glutamine (Gibco, UK), 100 μM 2‐mercaptoethanol (Gibco, UK) and 10^6^ leukaemia inhibitory factor (LIF) (Calbiochem, UK).^43^ They were incubated at 37°C at 95% O_2_, 5% CO_2_. Stem cells were passaged at a ratio of 1:8 at 80% confluence (approximately every 2 days) to maintain pluripotency. They were then used to plate on 0.1% gelatinized glass coverslips in 24 well plates for immunocytochemistry (Section 2.3) or in 60 mm nonadherent Petri dishes for neuronal differentiation (Section 2.2).

### 2.2 Neuronal differentiation from mES cells

mES were plated (Day 0) at 50,000 cells per/ml (0.5 * 10^6^) onto nonadherent 60 mm Petri dishes in Advanced DMEM‐F12:Neurobasal media (ADFNK) medium composed of advanced DMEM/F12–neurobasal (1:1) (Gibco, UK), 10% knockout serum replacement (KSR) (Gibco, UK), 1% P/S, 1% *L*‐glutamine (Gibco, UK), 100 μM 2‐mercaptoethanol (Gibco, UK) and maintained at 37°C in a 95% O_2_, 5% CO_2_, for the formation of EBs. The medium was changed on Day 2 and supplemented with 1 μM all‐trans RA/1 μM purmorphamine, which are known neutralizing agents. On Day 4 medium was changed back to ADFNK without RA or purmorphamine. On Day 6, EBs were washed in phosphate buffered saline (PBS), repelleted and resuspended in 5 ml of 0.25% trypsin–etheylenediaminetetraacetic acid (EDTA) (1x) on a rocker for 10 min at 37°C. Then, 5 ml of ADFNK was added to neutralize the trypsin–EDTA. The EB suspension was then triturated with a series of three progressively smaller in diameter fire‐polished glass Pasteur pipets, passed through a 70 μm cell strainer to remove large aggregates, centrifuged for 5 min at 180 rcf, resuspended in 5 ml ADFNB medium (advanced D‐MEM/F12: Neurobasal (1:1) (Gibco, UK), 1x B27 supplement (Invitrogen), 200 mM *L*‐glutamine (Gibco, UK), 1x Pen/Strep (Gibco, UK), 10 ng/ml Beta fibroblast growth factor (βFGF) (Gibco, UK), 10 ng/ml brain‐derived neurotrophic factor (BDNF) (Gibco, UK)) and counted using trypan blue exclusion. Individual cells were replated onto either 0.1% gelation or laminin‐coated (2 μg/cm^2^) coverslips (13 mm) at 200 cells/mm^2^ (30,000 cells) in 24‐well tissue culture plates and allowed to grow until D (day) 7, D14 and D21, with media changes every 2 days. This method of neuronal differentiation is a modification of the Peljto et al. protocol which has been shown to produce motor neurons.^44^

### 2.3 Immunocytochemistry

mES CGR8 cells at a density of 0.3 x 10^6^ were plated on gelatinised 13 mm coverslips in 24 well plates and fixed on Day 2 for 20 min in 4% paraformaldehyde (PFA) at room temperature (RT) for immunocytochemical analyses. Similarly, cultured differentiated neurons (mESn) (Section 2.2) were fixed in 4% PFA/RT for 20 min at RT at day 7, 14 and 21 of culture, as standard timepoints of examining neuronal maturation.^45^ Fixed cells were then permeabilized in 10% normal goat serum (NGS) (Fisher, UK) containing 0.1% Triton X‐100 (Sigma, UK) in PBS for 10 min at RT. For experiments where target antigens need to be visualised at the cell membrane, CellBrite cytoplasmic membrane dye (Biotium, UK) was incubated with fixed cells for 10 min at RT at the dilution shown in Table 2, prior to permeabilization. This timeframe is kept short so that the dye remains at the membrane and image analyses of target antigen (detected by antibody) localised with the membrane dye reveals the extent of colocalisation. After permeabilization, cells were washed again thrice for 5 min each in PBS, then incubated with primary and secondary antibodies (Table 1). Primary antibodies (Table 1) that detect the ERα‐66 and the mERs, ERα‐36 and GPER1, diluted in 10% NGS in PBS were applied to cells overnight on a shaker at 4°C. After three washes (5 min each) in PBS, cells were incubated with secondary antibodies (Table 1) in 10% NGS in PBS for 1 h. To detect the endoplasmic reticulum or the Golgi apparatus, we used stains that we could apply to fixed cells rather than available live stains (e.g., BoDIPY) to be compatible with prior antibody application. Stock solutions for concanavalin A conjugated to Alexa Flour 594 to detect the endoplasmic reticulum were prepared at 1 mg/ml in 0.1 M sodium bicarbonate and stored at −20°C. Then, 1 mg of Lectin HPA‐Alexa fluor 647 conjugate to detect the Golgi apparatus was dissolved in 1 ml of PBS and stored at −20°C. These organelle stains were then added at the concentrations and times shown in Table 2. After application of the organelle stain, cells were washed again thrice for 5 min each in PBS and coverslips mounted onto glass slides with Fluromount‐G mounting media with 4′,6‐diamidino‐2‐phenylindole (DAPI) (Invitrogen, UK) and sealed with DPX mountant (Sigma, UK). Slides were left to dry for at least 30 min before imaging cells on the microscope. Negative controls omitted the primary antibody/antibodies.

**TABLE 1:** Details of primary and secondary antibodies used for immunocytochemistry in mouse embryonic stem cell line (mES) and cultured differentiated neurons (mESn). Primary antibodies were bought from various sources and used at the dilution described at the table. Goat anti‐rabbit secondary antibodies conjugated to different fluorophores was used to obtain localisation of different antigens from the same coverslip

Antibody name/target/(cat no.)	Source	Host species	Dilution
ERα (STJ97499)	St. John's Laboratory, UK	Rabbit	1:300
GPER1 (STJ192629)	St. John's Laboratory, UK	Rabbit	1:300
ERα‐36 (ERA361‐1)	Alpha Diagnostics, USA	Rabbit	1:300
β‐tubulin‐III /neuronal marker (ab 18207)	Abcam, UK.	Mouse	1:300
MAP2/neuronal marker (AB_2313549)	Aves Lab, Davies, CA	Chicken	1:300
Neu‐N (Fox 3)/neuronal marker (AB_2313556)	Aves Lab, Davis, CA	Chicken	1:300
Goat anti‐rabbit IgG (H + L) Secondary antibody, DyLight 488 (11800074)	Invitrogen, Thermo‐Fisher Scientific, UK	Goat	1:300
Goat anti‐rabbit IgG (H + L) cross‐adsorbed secondary antibody, Alexa fluor 568 (A‐11011)	Invitrogen, Thermo‐Fisher Scientific, UK	Goat	1:300
Goat anti‐rabbit IgG (H + L) Cross‐Adsorbed secondary antibody, Alexa fluor 647 (19123672)	Invitrogen, Thermo‐Fisher Scientific, UK	Goat	1:300
Goat anti‐mouse IgG (H + L) Cross‐adsorbed secondary antibody, Alexa fluor 568 (A‐11004)	Invitrogen, Thermo‐Fisher Scientific, UK	Goat	1:300
Goat anti‐chicken IgY (H + L) Cross‐adsorbed secondary antibody, DyLight 350 (SA5‐10069)	Invitrogen, Thermo‐Fisher Scientific, UK	Goat	1:300
Goat anti‐chicken IgG (H + L) Cross‐adsorbed secondary antibody, Alexa fluor 647 (A‐21449)	Invitrogen, Thermo‐Fisher Scientific, UK	Goat	1:300

**TABLE 2:** Organelle and cell membrane stains used in experiments to determine subcellular localisation of the antigens in mouse embryonic stem cell line (mES) and cultured differentiated neurons (mESn). Stains were used for the times specified at the dilution specified either prior or after antibody application as detailed in Section 2

Antibody name/stain/(cat no.)	Source	Time (min)	Dilution/concentration
CellBrite cytoplasmic membrane dye (30022)	Biotium Inc, UK	10	1:200 in PBS
Concanavalin A, Alexa fluor 594 conjugate/endoplasmic reticulum marker (C11253)	Invitrogen, Thermo‐Fisher Scientific, UK	20	50 μg/ml
Lectin HPA From Helix pomatia (edible snail), Alexa fluor 647 conjugate/Golgi apparatus marker (L32454)	Invitrogen, Thermo‐Fisher Scientific, UK	20	5 μg/ml

### 2.4 Conjugation of antibodies with fluorophore

Since antibodies to the oestrogen receptor were raised in the same species, Anti‐ERα (Saint John's Laboratory, UK) and Anti‐GPER1 (Saint John's Laboratory, UK) were conjugated to fluorescein isothiocyanate (FITC) (Thermofisher, UK), as per the manufacturers’ protocols (Thermofisher UK). Briefly, anti‐ERα and anti‐GPER1 were initially concentrated using Amicon Ultra centrifugal filter units (Ultra 4 MWCO 30KDA) (Merck Millipore, UK) to generate stock solutions of concentrated Anti‐ERα and Anti‐GPER1 at 2 mg/ml in 0.1 M sodium bicarbonate. FITC stock solution was prepared at 1 mg/ml in anhydrous dimethyl sulphoxide (DMSO). Then, 50 μl of FITC solution was slowly add to the 2 mg/ml of antibody solution and incubated at 4°C for 1 h with continuous stirring. Free fluorophores were separated from conjugated antibody using PD‐10 column, Sephadex G‐25 M (BioRad, UK) and conjugated ERα/ FITC and GPER/FITC was eluted with PBS. In order to determine the labelling efficiency and concentration, absorbance values were measured at A280 and A495. For effective labelling, the degree of labelling should fall within 2–6 mol of FITC per 1 mol of antibody; typical concentrations were in the range of 0.2 mg/ml. The newly conjugated antibody was stored at −20°C until use.

### 2.5 Image analysis

Images were acquired using a Zeiss AxioImager Epiflourescent (Carl Zeiss MicroImaging GmbH) microscope with 20x objective under identical exposure times, gain and threshold with exposure times set by negative controls (no primary antibody added). Monochromatic images were analysed with EZcolocalisation (image processing plugin for Image J/Fiji, NIH Image)^46^ using methods previously described in.^47^ Since quadcolour experiments were possible, for mES cells that are homogenous, we analysed the presence of each oestrogen receptors in two or three different organelles in the same cell—that is, for example, ERα (green) in plasma membrane (red) and nucleus (DAPI blue) or ERα (green) in the nucleus (DAPI blue), endoplasmic reticulum (ConA‐red) and Golgi apparatus (HPA‐far red) in the same cell. However, not all cells in mESn cultures are neurons and must be identified by a neuron‐specific stain such as β‐III tubulin (far red) or neuronal nuclear protein (NeuN) (blue) that also stains the nucleus. Cells identified as neurons by these methods were then further analysed for the presence of oestrogen receptors in various organelles, using the EZcolocalisation (EZcoloc) plugin.

Manders coefficients determine the degree of overlap between fluorophores in a region delineated by the organelle stain even when signal intensities differ and this Ezcoloc plugin can be expanded to include more than two fluorophores. Hence, Manders coefficients M1 and M2 are pixel intensities, auto‐corrected for background, and describe the intercept of both fluorophores divided by either fluorophore 1 or fluorophore 2 whereby

$$\mathrm{Mx}=\frac{\sum_{i=1}^{n}N_{i,\text{coloc}}}{\sum_{i=1}^{n}N_{i}}$$

(see Figure S1 for details).^47^, ^48^ For our experiments, M1 was consistently used to denote the organelle/cellular stains, while M2 and M3 represented the oestrogen receptor antibodies, as denoted in the legends.

For ratio analyses of the distribution of target antigen between two organelles, organelle stains (Table 2) were used to identify the region of interest (ROI) in Fiji (NIH Image). The organelle outlines were overlaid on the channel containing the antigen of interest and mean intensity of the fluorescence signal in the nucleus, endoplasmic reticulum, Golgi apparatus and plasma membrane measured. For mES cells, between 45–50 cells were analysed for each target antigen localised at a particular site. For differentiated neurons (mESn), at least 20 neurons from each stage, that is, D7, D14, D21–positive for a neuronal marker (NeuN or βIII‐tubulin)–were analysed for each target antigen or at a particular subcellular site.

### 2.6 Statistical analysis

Neurons were analysed separately at different stages as D7, D14 and D21 and if there were no significant differences between days of differentiation, that is, D7, D14 or D21, neurons were combined across stages for ease of comparison to mES cells. All data are presented as mean ± SEM with graphs and statistics performed using Prism 9.0 (Graph Pad Software); numbers within columns or above columns refer to the number of cells analysed for each target antigen at a particular cellular location. Two‐way analysis of variance (ANOVA) followed by either Sidak's or Tukey's post hoc tests were used to compare between groups in experiments examining localisation of each ER within different organelles and for ratio analyses (Figure 4). For colocalisation of different ERs with each other in various organelles, differences between colocalisation in selected organelles was examined using Kruskal Wallis nonparametric test followed by the Dunn's post hoc test, since data was not normally distributed. In all cases, *p* < .05 was deemed statistically significant. Manders correlation coefficients ≥.5 were considered localised.^48^ Determination if colocalisation was statistically different (*p* < .05) from this threshold value was carried out using one sample *t* test and Wilcoxon's test.

## 3 RESULTS

### 3.1 Increase of ERα localisation in the nucleus and plasma membrane in mES‐derived neurons (mESn) compared to mES cells

**FIGURE 1:**
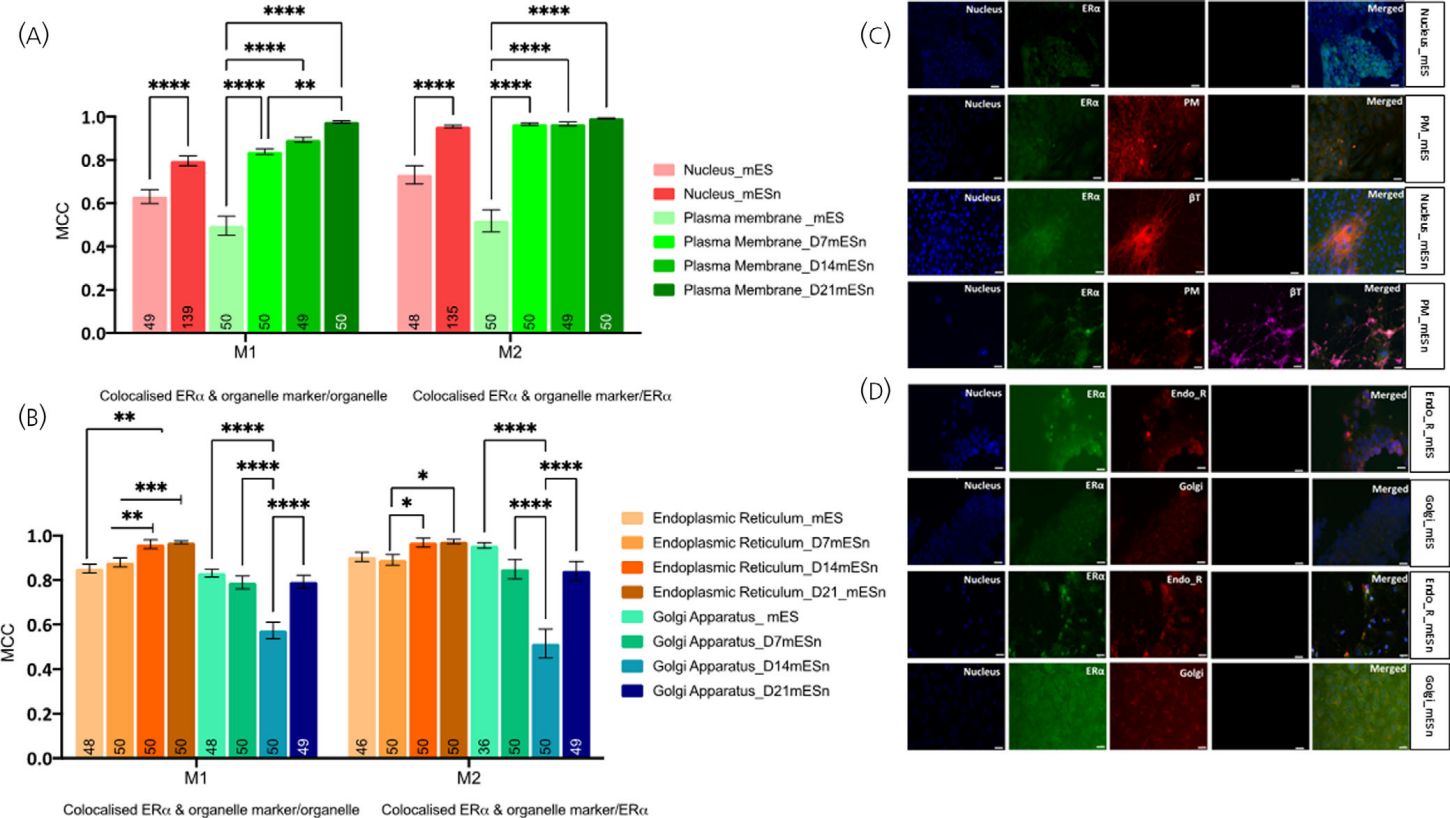
Subcellular localisation of ERα in mouse embryonic stem cell line (mES) and cultured differentiated neurons (mESn). Quantification of ERα‐66 in various organelles was carried out using image analyses using EZcoloc plugin (Fiji, NIH Image) to obtain Manders correlation coefficient (MCC) presented as M1 and M2 (Details in methods). MCC values ranging from 0 to 1 represent no colocalisation (0) or complete colocalisation (1) with 0.5 as the threshold for colocalisation (Figure S1). A two‐way ANOVA followed by Tukey's post hoc comparison compares between groups (A) Comparison of localisation of ERα in the nucleus and plasma membrane of mES and mESn. (B) Comparison of localisation of ERα in the endoplasmic reticulum and Golgi apparatus of mES and mESn. Data is presented as the mean ± SEM. (*n* = 36–48 mES, *n* = 49–139 mESn); number of analysed cells inside each bar. (C) Representative images (20x objective) of ERα (green), nuclear DAPI stain (blue), plasma membrane (red) in mES cells (first two rows). The third and fourth row show ERα (green), nuclear stain (blue), plasma membrane (red) and neuronal markers β‐tubulin (red or far red) in Day 14 mESn. (D) Representative images (20x objective) of ERα (green), nuclear DAPI stain (blue), endoplasmic reticulum or Golgi apparatus (red) in mES cells (first two rows). The third and fourth row show ERα (green), NeuN staining indicating neurons (blue), endoplasmic reticulum or Golgi apparatus (red) in Day 14 mESn. Scale bar, 100  μm. *p < .05, **p < .01; ***p < .001; ****p ≤ .0001, bT, βIII‐tubulin; EndoR, endoplasmic reticulum; Golgi, golgi apparatus; PM, plasma membrane.

**FIGURE 2:**
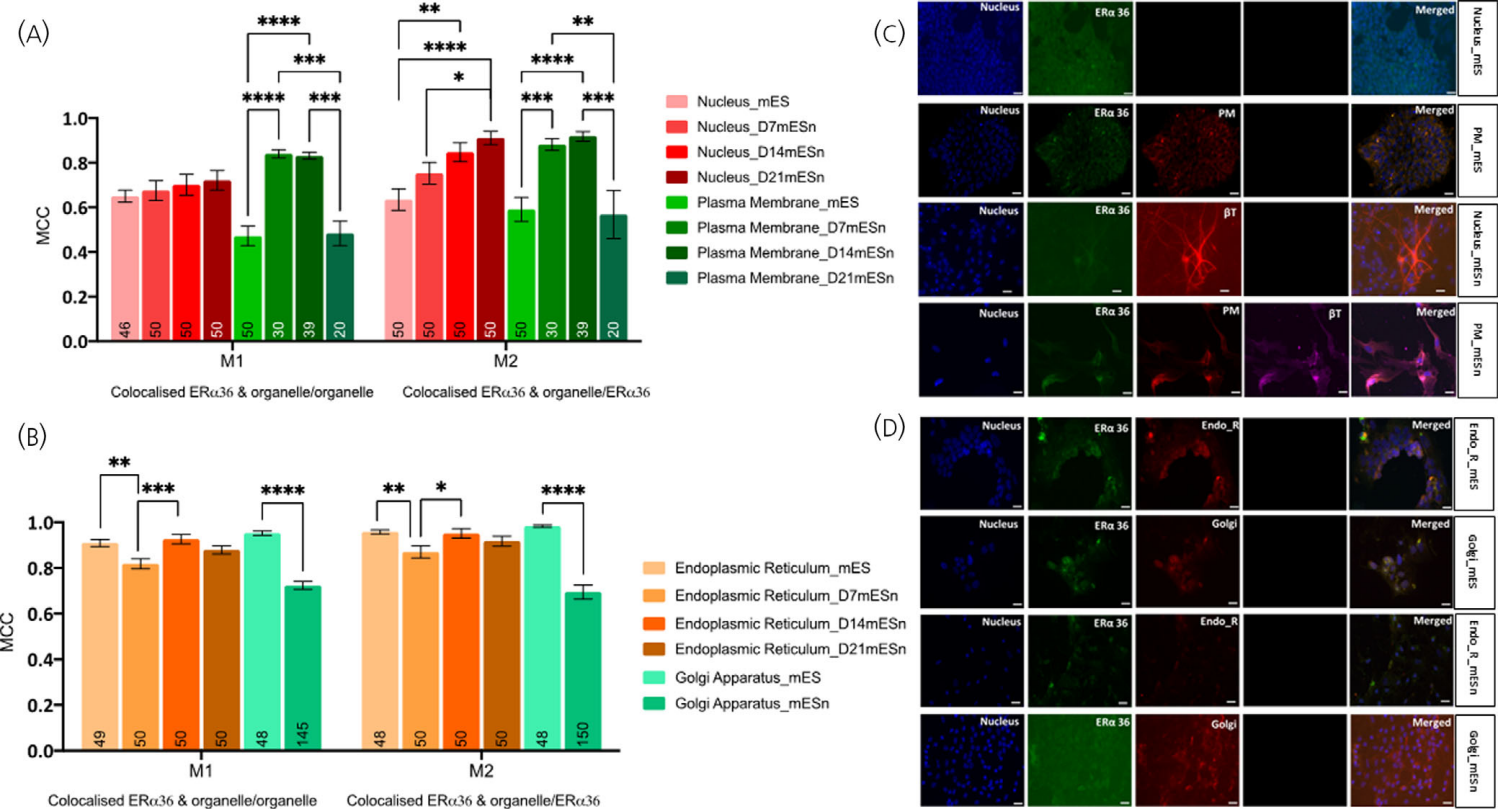
Subcellular localisation of ERα‐36 in mouse embryonic stem cell line (mES) and cultured differentiated neurons (mESn). Quantification of ERα‐36 in various organelles was carried out using image analyses using EZcoloc plugin (Fiji, NIH Image) to obtain Manders correlation coefficient (MCC) presented as M1 and M2 (details in Methods). MCC values ranging from 0 to 1 represent no colocalisation (0) or complete colocalisation (1) with 0.5 as the threshold for colocalisation (Figure S1). A two‐way ANOVA followed by Tukey's post hoc comparison compares between groups. (A) Comparison of localisation of ERα‐36 in the nucleus and plasma membrane of mES and mESn. (B) Comparison of localisation of ERα‐36 in the endoplasmic reticulum and Golgi apparatus of mES and mESn. Data is presented as the mean ± SEM. (*n* = 46–50 mES, *n* = 20–150 mESn); number of analysed cells is within each bar. (C) Representative images (20x objective) of ERα‐36 (green), nuclear DAPI stain (blue), plasma membrane (red) in mES cells (first two rows). The third and fourth row show ERα‐36 (green), nuclear stain (blue), plasma membrane (red) and neuronal markers β‐tubulin (red or far red) in Day 14 mESn. (D) Representative images (20x objective) of ERα‐36 (green), nuclear DAPI stain (blue), endoplasmic reticulum or Golgi apparatus (red) in mES cells (first two rows). The third and fourth row show ERα‐36 (green), NeuN staining indicating neurons (blue), endoplasmic reticulum or Golgi apparatus (red) in Day 14 mESn. Scale bar, 100  μm. *p < .05, **p < .01; ***p < .001; ****p ≤ .0001. bT, βIII‐tubulin; EndoR, endoplasmic reticulum; Golgi, golgi apparatus; PM, plasma membrane.

**FIGURE 3:**
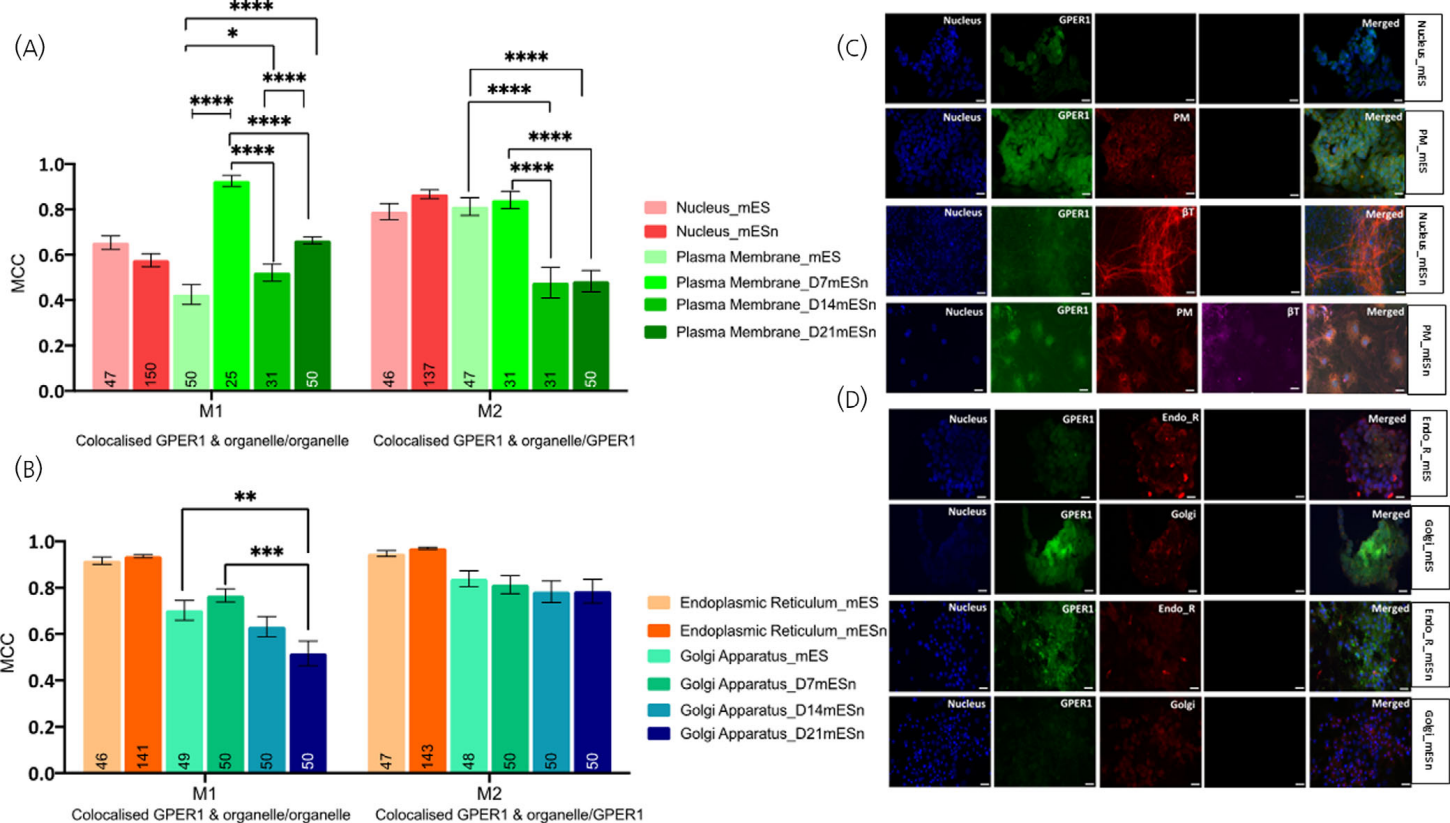
Subcellular localisation of GPER1 in mouse embryonic stem cell line (mES) and cultured differentiated neurons (mESn). Quantification of GPER1 in various organelles was carried out using image analyses using EZcoloc plugin (Fiji, NIH Image) to obtain Manders correlation coefficient (MCC) presented as M1 and M2 (Details in methods). MCC values ranging from 0 to 1 represent no colocalisation (0) or complete colocalisation (1) with 0.5 as the threshold for colocalisation (Figure S1). A two‐way ANOVA followed by Tukey's post hoc comparison compares between groups. (A) Comparison of localisation of GPER1 in the nucleus and plasma membrane of mES and mESn. (B) Comparison of localisation of GPER1 in the endoplasmic reticulum and Golgi apparatus of mES and mESn. Data is presented as the mean ± SEM. (*n* = 46–50 mES, *n* = 31–150 mESn); number of analysed cells is within each bar. (C) Representative images (20x objective) of GPER1 (green), nuclear DAPI stain (blue), plasma membrane (red) in mES cells (first two rows). The third and fourth row show GPER1 (green), nuclear stain (blue), plasma membrane (red) and neuronal markers β‐tubulin (red or far red) in Day 14 mESn. (D) Representative images (20x objective) of GPER1 (green), nuclear DAPI stain (blue), endoplasmic reticulum or Golgi apparatus (red) in mES cells (first two rows). The third and fourth row show GPER1 (green), NeuN staining indicating neurons (blue), endoplasmic reticulum or Golgi apparatus (red) in Day 14 mESn. Scale bar, 100 μm. *p < .05, **p < .01; ***p < .001; ****p ≤ .0001. bT, βIII‐tubulin; EndoR, endoplasmic reticulum; Golgi, golgi apparatus; PM, plasma membrane.

Since most studies have characterized ERα‐66 as a nuclear receptor with small traces being found in the cytoplasm and plasma membrane (reviewed in^13^), we first decided to determine the localisation of ERα in mES cells and mESn (Figure 1A–D), before proceeding to ERα‐36 (Figure 2A–D) and GPER1 (Figure 3A–D). For the purposes of these experiments, the M1 overlap coefficient is better suited to understand the localisation of the target antigen within each cellular compartment, since this denotes the level of antigen in that compartment.

ERα‐66 localises differentially in mES and mES derived neurons. Analysis of nuclear and membrane localisation revealed a significant increase in colocalisation in mESn in both these compartments compared to mES cells (Figure 1A,C). There are also higher levels of ERα‐66 colocalisation in the plasma membrane of D21 mESn compared to D7 mESn (Figure 1A; F(5, 757) = 87.14; *p* < .0001). Previous studies have localized minor amounts of ERα in the endoplasmic reticulum,^49^ but to date no study has quantified ERα in the Golgi apparatus. Results reveal high localisation of the ERα‐66 in both the endoplasmic reticulum and Golgi apparatus across both cell types. Although there are minor differences between mES and mESn in endoplasmic reticulum localisation of ERα‐66 (Figure 1B,D), there is a decrease in localisation of ERα in the Golgi apparatus in neurons at the Day 14 stage (Figure 1B,D; F(7,760) = 40.73, *p* < .0001).

### 3.2 Localisation of ERα‐36 and GPER1 in subcellular compartments is similar in mES and mESn cells

Both ERα‐36 and GPER1 are localised in the nucleus in mES and mESn (Figure 2A,C for ERα‐36; Figure 3A,C for GPER1) to a similar extent. For both these receptors, there is a transient increase in localisation in the plasma membrane from mES, where there is no appreciable localisation, to D7‐mESn. However, as differentiation of neurons proceeds, there is a decrease in plasma membrane localisation of both ERα‐36 and GPER1 to levels similar to those found in mES cells (Figure 2A; *F*(7, 658) = 17.30, *p* < .0001 and Figure 3A; *F*(5, 683) = 17.22, *p* < .0001). Although there are no differences in localisation of either of these receptors in the endoplasmic reticulum between mES and mESn, there is a decrease in localisation of these receptors in the Golgi apparatus as neuronal differentiation proceeds (Figure 2B; *F*(5, 776) = 38.95, *p* < .0001 and 2D for ERα‐36 and Figure 3B; *F*(5, 762) = 41.38, *p* < .0001 and Figure 3D for GPER1).

**FIGURE 4:**
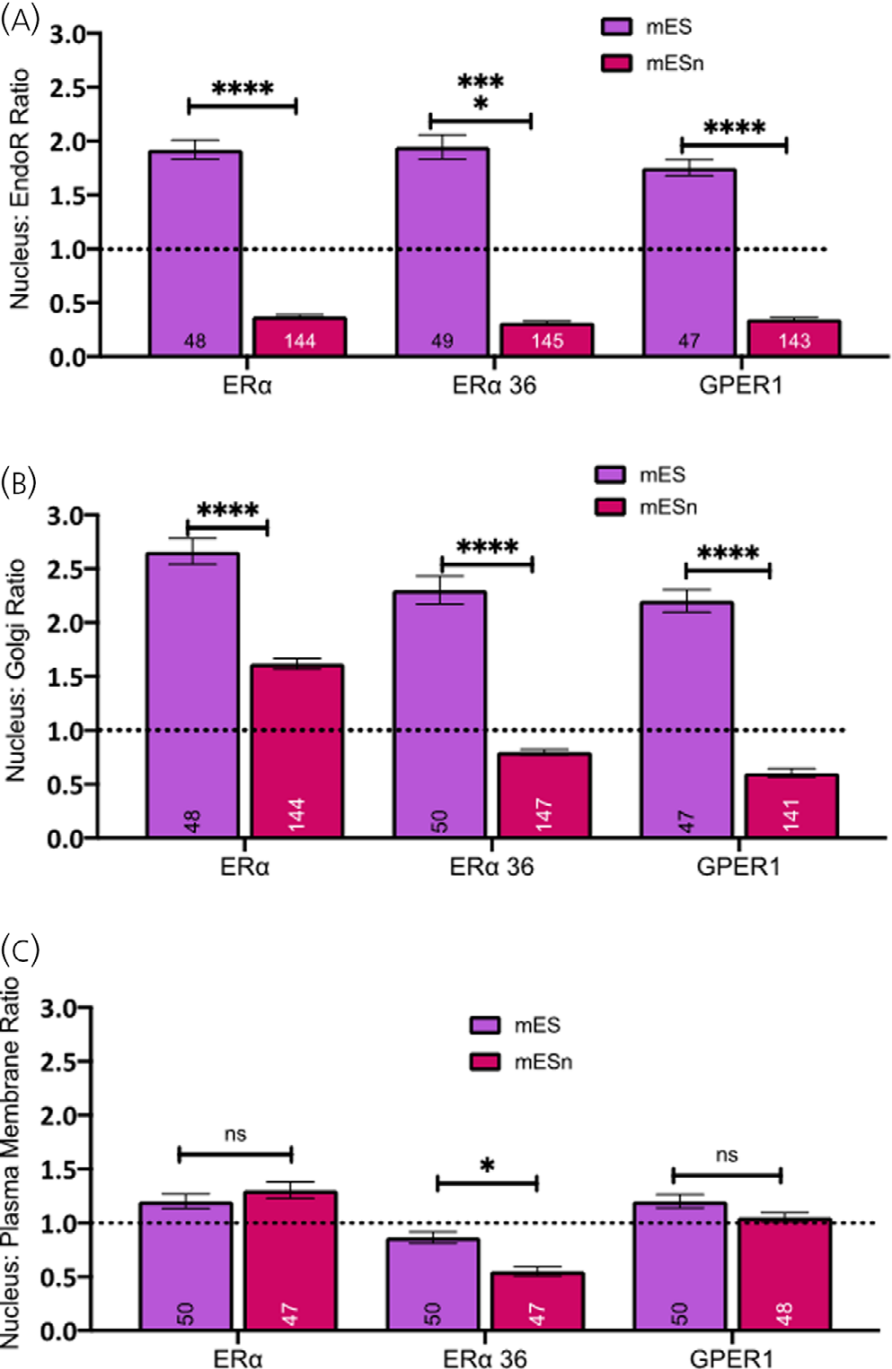
The distribution of oestrogen receptors in mouse embryonic stem cell line (mES) and cultured differentiated neurons (mESn) cells. (A) The distribution of ERα‐66, ERα‐36 and GPER1 in the nucleus versus the endoplasmic reticulum is shown by the nuclear: endoplasmic reticulum ratio in mES cells compared to mESn cells. (B) Similarly, the distribution of ERα‐66, ERα‐36 and GPER1 in the nucleus versus the Golgi apparatus is shown by the nuclear: Golgi apparatus ratio in mES cells compared to mESn cells. (C) The distribution of ERα‐66, ERα‐36 and GPER1 in the nucleus versus the plasma membrane is shown by the nuclear: plasma membrane ratio in mES cells compared to mESn cells. For (A) and (B), mESn represents data, combined from all stages of neuronal differentiation while for (C), mESn represents data from Day 7 differentiated neurons only. *p < .05; ****p ≤ .0001. No of analysed cells are given within each bar. Two way ANOVA followed by Sidak's multiple comparison test compares between groups.

The relative levels of the oestrogen receptors between the nucleus and the other organelles (plasma membrane, endoplasmic reticulum, Golgi apparatus) is shown in Figure 4A–C. As can be seen, there are higher relative levels of all the oestrogen receptors in the nucleus as compared to the endoplasmic reticulum (Figure 4A; *F*(1, 570) = 2043, *p* < .0001). Although there are higher levels of ERα‐66 in the nucleus compared to the ERα‐66 in the Golgi apparatus in both mES and mESn cells (Figure 4B; *F*(1, 571) = 619.9, *p* < .0001), there are lower levels of ERα‐36 and GPER1 in the nucleus compared to the Golgi apparatus of mESn cells compared to mES cells (Figure 4B). As differentiation proceeds, the level of ERs in the nucleus decrease as they rise in the endoplasmic reticulum and the Golgi apparatus. However, levels of ERα‐66, ERα‐36 or GPER‐1 are roughly equally distributed between nucleus and plasma membrane in mES cells and this does not change in mESn for either ERα or GPER1 (Figure 4C; *F*(1, 286) = 6.242, *p* = 0.0130). For ERα‐36, there is more protein at the plasma membrane in mESn than in mES cells, compared to levels in the nucleus (Figure 4C).

### 3.3 Oestrogen receptors are present in the same organelle but are differentially distributed in mES and mESn

**FIGURE 5:**
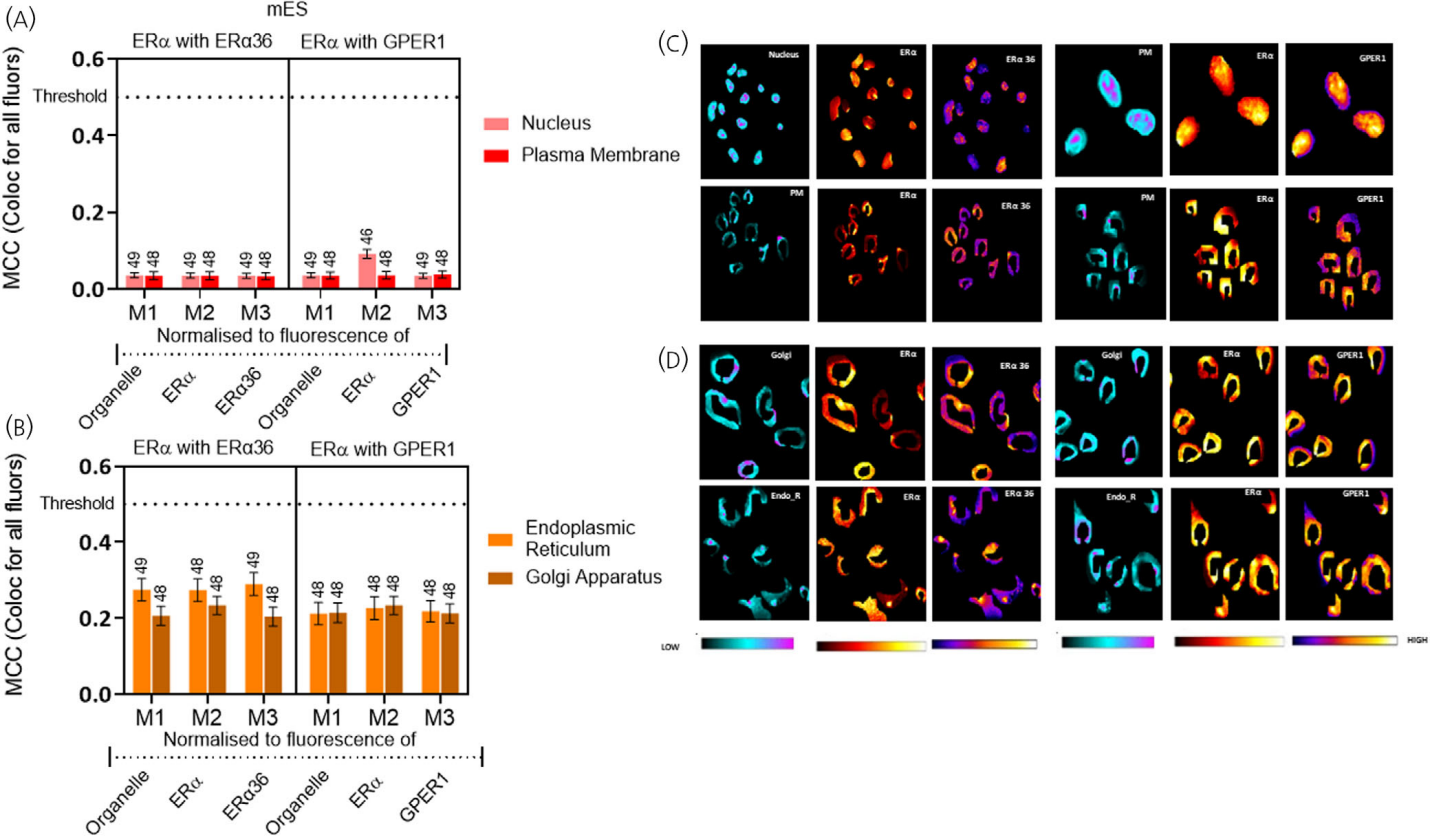
Colocalisation of ERα‐66 with ERα‐36 and GPER1 in mouse embryonic stem cell line (mES) cells. Quantification of colocalisation between ERα‐66 and each of the other oestrogen receptors that is, ERα‐36 and GPER1 was carried out using Manders correlation coefficient (MCC) presented as M1, M2, and M3, whereby M1 values represent intersection of all fluorophores in the organelle, M2 values represent the intersection of all fluorophores in ERα‐positive pixels and M3 represent the intersection of all fluorophores in either ERα 36 or GPER1‐positive pixels. MCC values ranging from 0 to 1 represent no colocalisation (0) or complete colocalisation (1) with 0.5 as the threshold for colocalisation (Figure S1). (A) Comparison of colocalisation of ERα‐36 (left) and GPER1 (right) with ERα‐66 in the nucleus and plasma membrane of mES. (B) Comparison of colocalisation comparison of ERα‐36 (left) and GPER1(right) with ERα‐66 in the Golgi apparatus and endoplasmic reticulum of mES. Data is presented as the mean ± SEM. (C) Heatmaps of ERα‐36 (left) and GPER1 (right) quantifying the amount of each antigen in nucleus (first row) and plasma membrane (second row). (D) Heatmaps of ERα‐36 (left) and GPER1 (right) quantifying the amount of each antigen in the endoplasmic reticulum and Golgi apparatus. All MCC values are significantly below 0.5 MCC (dotted line; Appendix S1 1A). EndoR, endoplasmic reticulum; Golgi, Golgi apparatus; PM, plasma membrane. The number of cells analysed is given above each bar.

**FIGURE 6:**
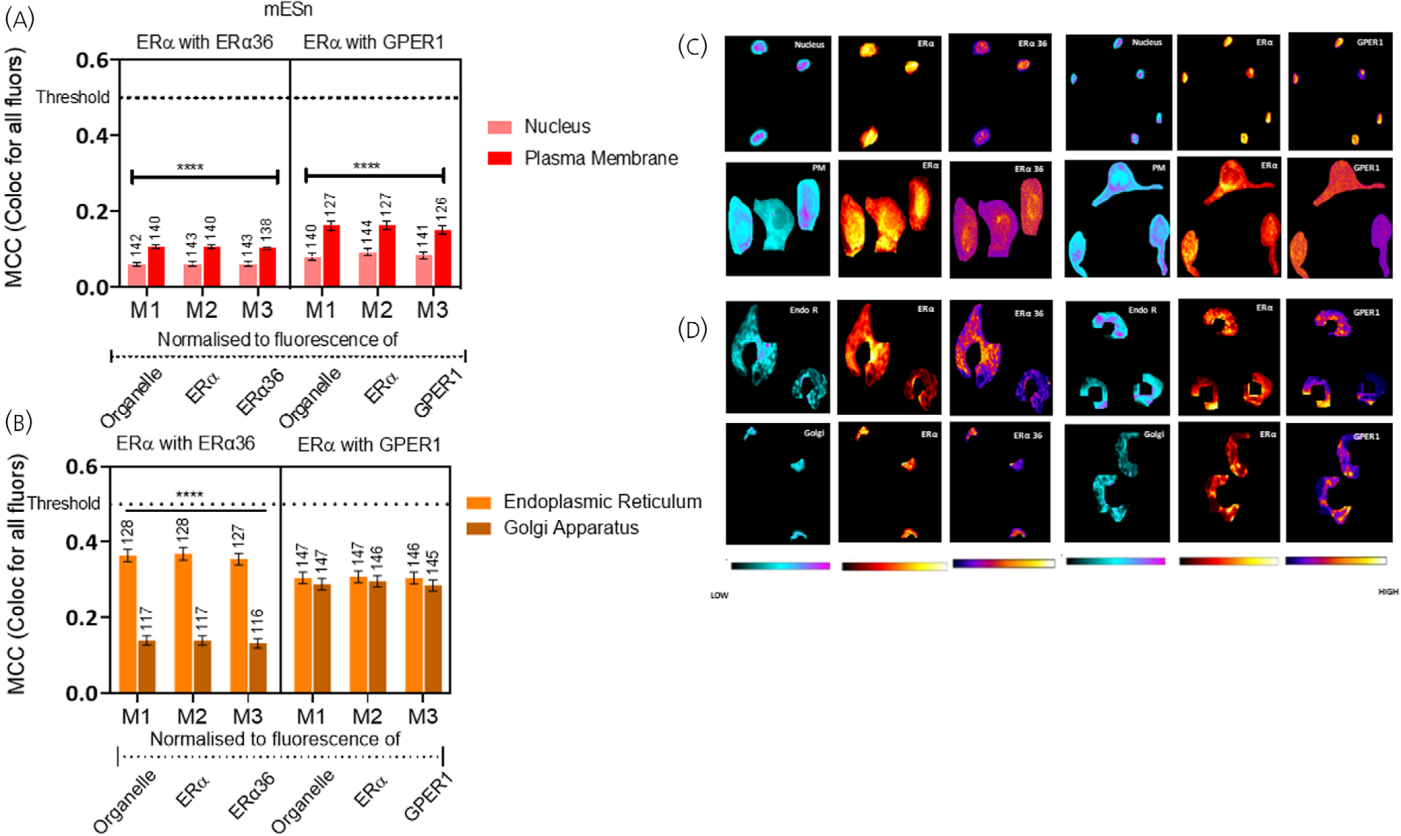
Colocalisation of ERα‐66 with ERα 36 and GPER1 in neurons derived from mouse embryonic stem cell line (mES) cells (cultured differentiated neurons [mESn]). Quantification of colocalisation between ERα‐66 and each of the other oestrogen receptors that is, ERα‐36 and GPER1 was carried out using Manders correlation coefficient (MCC) presented as M1, M2, and M3, whereby M1 values represent intersection of all fluorophores in the organelle, M2 values represent the intersection of all fluorophores in ERα‐positive pixels and M3 represent the intersection of all fluorophores in either ERα 36 or GPER1‐positive pixels. MCC values ranging from 0 to 1 represent no colocalisation (0) or complete colocalisation (1) with 0.5 as the threshold for colocalisation (Figure S1). (A) Comparison of colocalisation of ERα‐36 (left) and GPER1 (right) with ERα‐66 in the nucleus and plasma membrane of mESn. (B) Comparison of colocalisation comparison of ERα‐36 (left) and GPER1(right) with ERα‐66 in the Golgi apparatus and endoplasmic reticulum of mES. Data is presented as the mean ± SEM. (C) Heatmaps of ERα‐36 (left) and GPER1 (right) quantifying the amount of each antigen in nucleus (first row) and plasma membrane (second row). (D) Heatmaps of ERα‐36 (left) and GPER1 (right) quantifying the amount of each antigen in the endoplasmic reticulum and Golgi apparatus. Day 14 neurons are shown in (C) and (D). ****p ≤ .0001, cf. the other organelle in the same MCC group using nonparametric Kruskal Wallis test followed by Dunn's post hoc comparison between groups. For ERα colocalised with ERα‐36, the Kruskal Wallis test shows a statistically different median between colocalisation in the nucleus versus the membrane (H (5) = 148.7, *p* < .0001) and between the endoplasmic reticulum and the Golgi apparatus (H (5) = 251.2, *p* < .0001). For ERα colocalised with GPER1, the Kruskal Wallis test shows a statistically different median between colocalisation in the nucleus versus the membrane (H (5) = 123.5, *p* < .0001). All MCC values are significantly below 0.5 MCC, the threshold for colocalisation (dotted line; Appendix S1 1B). EndoR, endoplasmic reticulum; Golgi, Golgi apparatus; PM, plasma membrane. The number of cells analysed is given above each bar.

**FIGURE 7:**
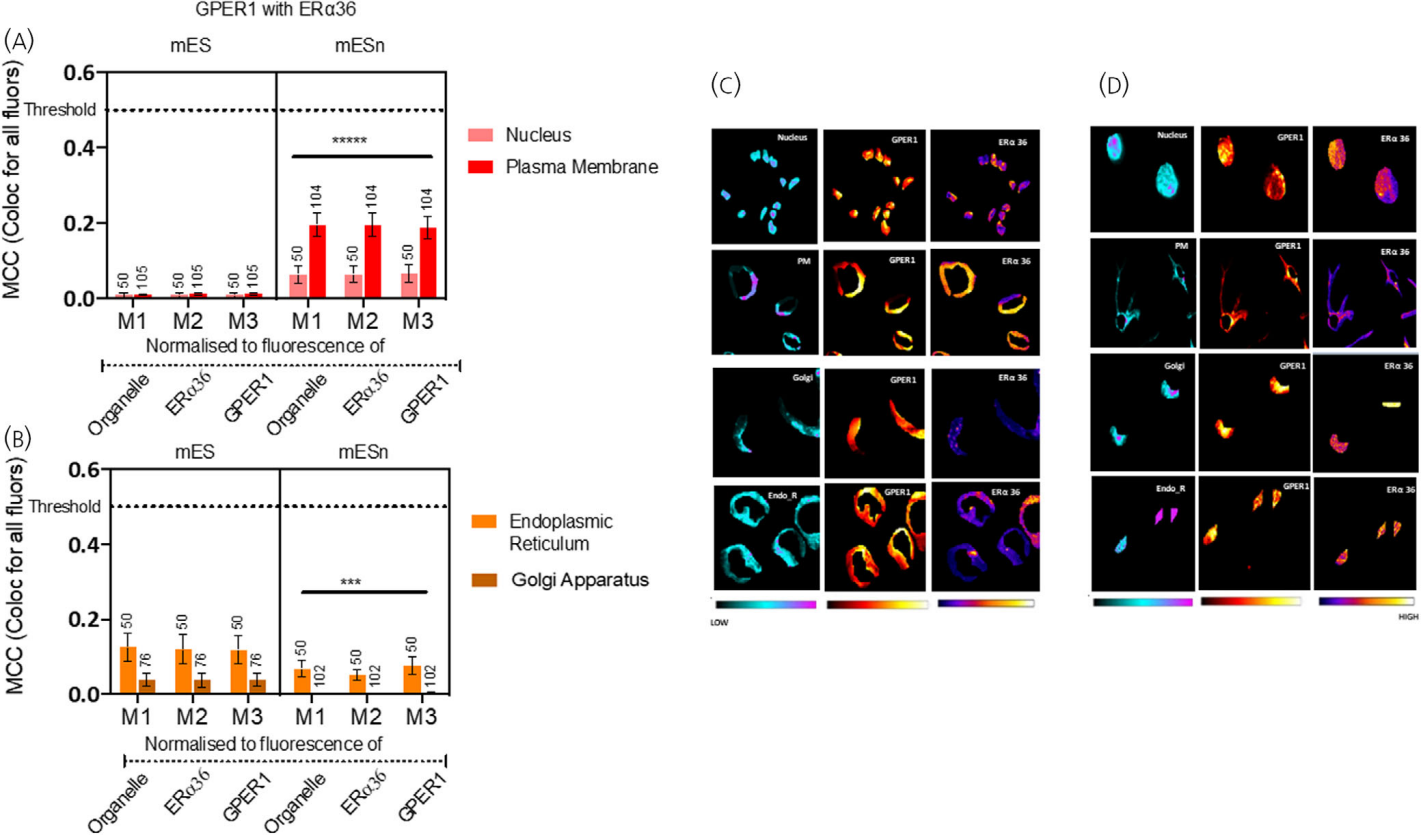
Colocalisation of ERα‐36 with GPER1 in mouse embryonic stem cell line (mES) and neurons derived from mES (mESn). Quantification of colocalisation between ERα‐36 and GPER1 was carried out using Manders correlation coefficient (MCC) presented as M1, M2, and M3, whereby M1 values represent intersection of all fluorophores in the organelle, M2 values represent the intersection of all fluorophores in GPER1‐positive pixels and M3 represent the intersection of all fluorophores in ERα‐36‐positive pixels. MCC values ranging from 0 to 1 represent no colocalisation (0) or complete colocalisation (1) with 0.5 as the threshold for colocalisation (Figure S1). (A) Colocalisation of ERα‐36 and GPER1 in the nucleus and plasma membrane of mES (left panel) and mESn (right panel). (B) Colocalisation of ERα‐36 and GPER1 in the Golgi apparatus and endoplasmic reticulum of mES (left panel) and mESn (right panel) (C) Heatmaps of ERα‐36 and GPER1 quantifying the amount of each antigen in nucleus and plasma membrane (first two rows) and in Golgi apparatus (third row) and endoplasmic reticulum (fourth row) in stem cells (mES). (D) Heatmaps of ERα‐36 and GPER1 quantifying the amount of each antigen in nucleus and plasma membrane (first two rows) and in Golgi apparatus (third row) and endoplasmic reticulum (fourth row) in neurons derived from mES cells (mESn). ****p < .001 cf. the other organelle in the same MCC group using nonparametric Kruskal Wallis tests followed by Dunn's post hoc test. For ERα‐36 and GPER1 colocalised in mES cells, there is no statistically significant difference in colocalisation between nucleus and plasma membrane as determined by post hoc tests (H (5) =12.21; *p* = .0320). For ERα‐36 and GPER1 in mESn cells, there is significantly increased colocalisation in plasma membrane than in nucleus (H (5) = 150.8; *p* < .0001). For ERα36 and GPER1 in mESn cells, there is significantly more colocalisation in the endoplasmic reticulum than the Golgi apparatus (H (5) = 50.09; *p* < .0001). All values are significantly below the threshold of 0.5 MCC (dotted line; Appendix S1 1C). EndoR, endoplasmic reticulum; Golgi, Golgi apparatus; PM, plasma membrane. The number of cells analysed is given above each bar.

Our data so far reveals localisation of oestrogen receptors in all four subcellular compartments; that is, nucleus, endoplasmic reticulum, plasma membrane and Golgi apparatus. Do these receptors colocalise with each other? There is low colocalisation (<0.5) of ERα and the variant ERα‐36 or ERα and GPER1 within mES cells, independent of compartment (Figure 5A,B; Appendix S1 1A) To evaluate this, we used heat maps to observe differential distribution of these receptors within an organelle (Figure 5C,D). Similar patterns were observed in the mESn where there was no colocalisation in nucleus or plasma membrane (Figure 6A; Appendix S1 1B and 6C) or endoplasmic reticulum or Golgi apparatus (Figure 6B; Appendix S1 1B and 6D) for ERα/ERα‐36 or ERα/GPER1. We next investigated the colocalisation of the two membrane ERs, that is, ERα‐36 with GPER1. Independent of organelle, there was very little colocalisation of these two receptors in either mES (Figure 7A–C) or mESn (Figure 7A–D). Therefore, despite the presence of these oestrogen receptors capable of rapid nongenomic signalling in each organelle, they appear to occupy distinct spaces within the organelle, independent of neuronal development.

## 4 DISCUSSION

In the present study, we used two cell types, the mouse embryonic stem cells (mES) CGR8 and differentiated neurons, both of which express ERα‐66, ERα‐36 and GPER1.

Our data reveal three findings—namely (1) ERα‐66 localisation in both nucleus and membrane increases as neuronal maturation proceeds (2) GPER1 and ERα‐36 localise similarly—that is, in the nucleus and endoplasmic reticulum independent of stem cell or neuronal status with transient increases in the plasma membrane and decreases in the Golgi apparatus as neuronal maturation proceeds (3) ERα‐66, ERα‐36 and GPER1 are not colocalized with each other in any subcellular compartment despite the presence of all receptors in these compartments. Finally, for the first time we identify and characterise ERα‐36 localisation in mES derived neurons in four subcellular compartments visualised that is, the nucleus, plasma membrane, endoplasmic reticulum and Golgi apparatus.

### 4.1 Presence of ERα‐66 and mERs in stem cells

One of our objectives was to provide a model neuronal system for the study of oestrogen receptor signalling, in particular for rapid, nongenomic signalling. We used the CGR8 mES cell line, which does not require a feeder layer to sustain pluripotency. Instead, stemness is maintained with the addition of LIF in the media. With this protocol, we generated βIII‐tubulin positive cells by D7 (13 days total). This is one of the fastest and simplest methods for neural differentiation available since unlike other methods, we do not need to seed the generated EBs on adherent plates or harvest neural rosettes, which are additional complex steps often used.^50^ A similar technique to ours has been employed recently by Hanafiah et al. to obtain NPCs by Day 8 and neurons by Day 12, as demonstrated via the expression of nestin and neurofilament markers.^51^ In our protocol, we obtain NPCs by Day 6 and supplement BDNF and βFGF in the neuronal media to ensure neuronal maturation over the following 3 weeks. Although the three selected timepoints for neuronal characterization (D7, D14 and D21) do not correspond to any specific embryonic or postnatal developmental stage, in the vast majority of neuronal studies that contain a developmental element, characterization is carried out within this timeframe.^45^, ^53^, ^54^, ^55^

Our data clearly show the presence of all receptors in the mES and in neurons derived from mES. Human ES and EBs express both ERα‐66 and ERβ^55^; however, ERβ is not reliably detectable with currently available antibodies and hence not assayed in this study.^56^ GPER1 has been recently shown to be expressed by neural stem cells in the rat embryo^57^ but to the best of our knowledge, neither GPER1 or ERα‐36 has not been demonstrated in the mES. Furthermore, no previous study has shown the subcellular localisation of these receptors within mES, or neurons derived from stem cells. (Section 4.2).

### 4.2 Subcellular localisation of the ERα, GPER1 and ERα‐36 in mES or in neurons derived from mES

#### 4.2.1 Localisation at the ERs in the plasma membrane

Our study shows that only ERα‐66 is reliably present in the plasma membrane of mES. In mES, a membrane limited conjugate E2‐BSA increases cell motility and F‐actin in a Src‐EGFR dependent manner, suggesting that rapid signalling from the membrane has functional relevance, although the receptor mediating this was not identified.^58^ Our data suggests that this is most likely ERα. In a hypothalamic cell line, mHypo‐38^59^ and in neurons^60^ and astrocytes,^61^ ERα‐66 levels at the plasma membrane are increased by 17β‐oestradiol, rapidly within 30 min. This shows that this full length ERα‐66 can be targeted to the plasma membrane in neurons. Similarly, although there is low localisation of GPER1 and ERα‐36 (Manders correlation coefficient [MCC] < 0.5) in the plasma membrane of mES, plasma membrane localisation increases for all oestrogen receptors as neuronal maturation proceeds, suggesting that GPER1 and ERα‐36 can also be tethered to the membrane. In SKBR3 breast cancer^62^ and human embryonic kidney (HEK) cells,^63^ around 50% of the ERα‐36 is at the membrane where it increases ERK signalling rapidly similar to GPER1‐induced EGFR‐dependent ERK signalling^25^; tethering at the membrane thus may provide an opportunity to access growth factor receptors. Rapid signalling by ERK due to activation of growth factors is linked to maturation of neurons from stem cells^65^, ^66^ and it is possible that increased plasma localisation during neuronal maturation for the classical ERα‐66 as well as the mERs in this study may result in increased nongenomic signalling by crosstalk with growth factor receptors.

#### 4.2.2 Localisation of the ERs in the nucleus

Our results also show that although all oestrogen receptors are present in the nucleus, ERα‐66 uniquely amongst the three oestrogen receptors increases its localisation in the nucleus, as maturation proceeds. 40% of the ERα‐36 variant has been shown in the nucleus in HEK cells, where it can act as a dominant negative mutant and decrease transcriptional activation by ERα‐66 from a luciferase reporter.^63^ Since nuclear ERα‐66 increase but levels of nuclear ERα‐36 and GPER1 remain the same as neurons differentiate, it is possible that transcriptional signalling by ERα‐66 increases as neuronal maturation proceeds. However, it is important to note that despite high localisation of every oestrogen receptor in the endoplasmic reticulum, nucleus and Golgi apparatus in mES cells, the distribution of all oestrogen receptors appears to be nucleus‐biased (Figure 4) and may reflect studies that show that global transcriptional activity is higher in stem cells than in differentiated cells.^67^, ^68^

In breast cancer cells, the majority of GPER1 appears to be intracellular with minor amounts on the cell membrane^68^; in COS cells, it is primarily seen in the endoplasmic reticulum where it mediates calcium release in response to oestrogen.^26^ The distribution of GPER1 in mES cells where it appears to show significant nuclear localisation is unusual but not unreported. Also, other G‐protein coupled receptors such as muscarinic acetylcholine receptors,^69^ adrenergic receptors^70^ and endothelin receptors^71^ can localise in the nucleus in multiple cell lines across various species^73^, ^74^ and nuclear translocation of GPER1 with a single nucleotide polymorphism (SNP) in cancer associated fibroblasts increases the migration of neighbouring cells.^75^, ^76^ Although deglycosylation of this mutant GPER1 increases GPER1 binding to chromatin and transcription from the *c‐fos* promoter,^75^ the function of wildtype GPER1 in the nucleus and perinuclear compartments^76^ remains elusive.

Our data show that ERα‐66 and GPER1 are equally distributed between the nucleus and plasma membrane in mES cells or in differentiated neurons, suggesting that both nongenomic and genomic signalling occur. Our data also suggest that as neuronal differentiation proceeds, ERα‐66 is the dominant player driving transcription in the nucleus, but all oestrogen receptors decrease their nuclear localisation and redistribute to the other cellular compartments such as the plasma membrane to possibly increase nongenomic signalling. In support, ERα‐36 distribution shows a decrease in nuclear localisation and movement towards the membrane (Figure 4C).

### 4.3 There is no colocalisation amongst receptors in any subcellular compartment

The heatmap distribution (Figures 5, 6, 7) shows that despite the presence of ERα‐66, ERα‐36 and GPER1 in all cellular compartments studied, they do not colocalise, with MCC values in the range of 0 to 0.3. What are the reasons for this further spatial organisation into microdomains? Functional microdomain organisation of receptors into caveolae has been suggested by the interaction of full length ERα with different CAV isoforms. For example, CAV1 allows ERα to couple to G*α*_*q*_ via mGluR5 in the striatum but via mGluR1A in the arcuate hypothalamus and hippocampus, to activate ERK signalling. However, in the dorsal root ganglion, ERα coupling to CAV3 may allow for interaction with mGluR2/3 to activate Gα_i_ and subsequent reduction in protein kinase A signalling.^16^ Although GPER1 coupling to CAV has not been shown in the CNS, this separation into microdomains could allow ERα‐66 or ERα‐36, where colocalisation with CAV is seen in Hec1 cells,^24^ to activate Gα proteins.

Our previous review^31^ suggested several scenarios in which GPER1 could interact with ERα‐66, with the focus on GPER1 being a “collaborator”.^77^ One scenario is to increase the level of a convergent output by using two different signalling pathways and separating these receptors into microdomains within these subcellular compartments may allow for access to these different pathways. For example, calcium increase in COS cells is mediated by both ERα‐66 and GPER1 in response to oestrogen but ERα‐66 uses a phospho‐lipase C‐dependent mechanism whereas GPER1 uses a EGFR‐mediated mechanism.^26^ Hence, calcium increases are large in this cell line and are due to both receptors acting via spatially distinct pathways in an additive manner. In the cortex, where ERα‐66 and insulin growth factor receptor interaction mediates neuroprotection via inhibition of GSKβ,^79^, ^80^ GPER1 achieves neuroprotection via activation of death activated protein kinase 1 (DAPK1),^80^ suggesting again that various signalling pathways could be used by differently located oestrogen receptors to facilitate a common cellular endpoint.^81^ Spatial separation within subcellular compartments may also be a way to achieve either independent outputs or sequential activation from each receptor. For example, mGluR1‐mediated long‐term depression (LTD) and synapse silencing by oestrogen in the hippocampal CA3 is via GPER1, independent of ERα and ERβ although all these receptors are expressed in this region.^82^ Coupled signalling where activation of one receptor leads to the regulation of the other may also be a consequence of unique compartmentalisation. For example, priming of lordosis or female sex behaviour by GPER1, acting as a “gain amplifier" at a cytoplasmic location may be coupled to ERα‐mediated activation of transcription in the nucleus by an intervening signal transduction cascade.^82^, ^84^ Although GPER1 and ERα‐36 are implicated predominantly in nongenomic signalling, they are not colocalised in either mES or mESn. Both receptors bind a plethora of ligands, including some aromatic plant compounds (GPER1),^81^ 17α‐oestradiol, oestriol, oestrone (ERα‐36),^84^ with a wider spectrum of ligand specificity than full length ERα and spatial separation in the same organelle may also be a way to allow for discrete access to different ligands.

## 5 CONCLUSION

Most studies in this field detail the translocation of nuclear receptors, in the presence of some typical stimulus, such as endogenous ligand or antagonist but do not explore colocalisation. Our results, in an accessible system of differentiated neurons from embryonic stem cells show, for the first time, expression of these endogenous oestrogen receptors in different subcellular compartments and demonstrates, at least in this cell system, that colocalisation appears to be low. Currently, the mechanisms or reasons for such differential localisation are unclear and have not been explored for most nuclear hormone receptors. The distribution of GPER‐1 has also been controversial with some studies showing predominantly localisation in the endoplasmic reticulum, or in the perinuclear space or in the cell membrane in a possibly cell‐specific manner^81^ (and references therein). Our data show that many subcellular locations are possible with the function of nuclear localisation of this G‐protein coupled receptor (GPCR) unknown.

Quantitative protein colocalisation for biomarkers, including nuclear hormone receptors and mERs is now being explored for more precise breast cancer therapy.^86^, ^87^ Our study supports the contention that such compartmentalisation and colocalisation analyses is, as argued by some other investigators,^87^ a field ripe for investigation since it is relevant to biological function.

## AUTHOR CONTRIBUTIONS

**DeAsia Davis:** Data curation; formal analysis; investigation; writing – original draft. **Ruby Vajaria:** Data curation; formal analysis. **Evangelos Delivopoulos:** Methodology; supervision; writing – original draft; writing – review and editing. **N Vasudevan:** Conceptualization; formal analysis; investigation; project administration; supervision; writing – original draft; writing – review and editing.

## CONFLICT OF INTEREST

The authors declare that they have no conflict of interest.

### PEER REVIEW

The peer review history for this article is available at https://publons.com/publon/10.1111/jne.13220.

## Supporting information


**APPENDIX S1.** Supporting Information.

**FIGURE S1.** An example workflow showing the intercept of different fluorophores (red, green, blue) and how colocalisation of these leads to the calculation of Manders correlation coefficients (M1, M2 or M3). For M3, the overlapping pixels for all three fluorophores is used as the numerator (C). In Figures 1, 2, 3, we sought to determine if ERs were present in different organelles. M1 always denoted the organelle stain while M2 denoted the specific oestrogen receptor antibody (ERα‐66, GPER1 and ERα‐36). Figure S1A is an example of these type of experiments If we wanted to determine if ERα was at the membrane, M1 would represent all overlapping pixels for the membrane stain and ERα as the numerator divided by the denominator that is, all pixels of the membrane stain. M2, on the other hand, would represent all overlapping pixels for the membrane stain and ERα as the numerator divided by all the pixels of ERα. If every pixel stained by the membrane stain also stained for ERα, M1 would be 1 and would represent complete colocalisation. If no pixel stained by the membrane stain overlapped with ERα staining, the numerator would be 0 and M1 would be zero.

## Data Availability

Data supporting the results in this paper are openly available at the Zenodo Repository at the doi:10.1186/s13058-021-01475-y.
