# Supplementary material for: Localisation of oestrogen receptors in stem cells and in stem cell‐derived neurons of the mouse
Source: J Neuroendocrinol. 2022 Dec 12;35(2):e13220. doi: 10.1111/jne.13220 (PMC10909416; doi:10.1111/jne.13220)
Supplement: Supplementary file 1 — APPENDIX S1. Supporting Information. [file JNE-35-e13220-s002.docx]

Supplementary file

These are statistics for Figure 5-7.

**Supplementary data 1A**: Statistical reporting for colocalisation of target antigens in different organelles. M1, M2 and M3 values obtained for colocalisation of different ERs in different values were compared to the MCC threshold of 0.5, indicating colocalisation, using the one sample t-test and Wilcoxon's test (Graph Pad Prism, CA). p values obtained in these tests that are <0.5 indicate that colocalisation is signifcantly different from the threshold value of 0.5.

For Figure 5A in mES cells, colocalisation of ERα-66 with ERα-36 in the nucleus and plasma membrane, reflected by M1, M2 and M3 values is significantly lower from the threshold of 0.5. Hence, Wilcoxon's test for M1 (nucleus) gives t(48)=67.77, p<0.0001 and for M1(plasma membrane) gives t(47)=45.38, p<0.0001. For M2 (nucleus), the value is t (48)=68.20, p<0.0001 while for M2 (plasma membrane), the value is t(47)=44.56, p<0.0001. For M3 (nucleus), the value is t(48)=69.45, p<0.0001 and for M3 (plasma membrane), the value is t (47)=53.63, p<0.0001. For Figure 5A in mES cells, colocalisation of ERα-66 with GPER1 in the nucleus and plasma membrane, reflected by M1, M2 and M3 values is significantly lower from the threshold of 0.5. Hence, for M1 (nucleus), t(48)=29; p<0.0001 and M1 (plasma membrane), the value is t(47)=51.62, p<0.0001. For M2 (nucleus), the value is t(45)=35.34, p<0.0001 and for M2 (plasma membrane), the value is t (47)=49.39, p<0.0001. Similarly, for M3 (nucleus), Wilcoxon's test reveals a value of t(48)=28.36, p<0.0001 while for M3 (plasma membrane), the value is t(47)=48.03, p<0.0001.

For Figure 5B in mES cells, colocalisaton of ERα-66 with ERα-36 in the endoplasmic reticulum and Golgi apparatus, reflected by M1, M2 and M3 values is significantly lower from the threshold of 0.5. Hence, Wilcoxon's test for M1 (endoplasmic reticulum), the value is t(48)=7.621, p<0.0001 and M1 (Golgi apparatus), the value is t(47)=11.64, p<0.0001. For M2 (endoplasmic reticulum), the value is t(47)=7.671, p<0.0001 and for M2 (Golgi apparatus), the value is t(47)=11.09, p<0.0001. Similarly, for M3 (endoplasmic reticulum), the value is t(48)=6.957, p<0.0001 and for M3(Golgi apparatus), the value is t(47)=11.91, p<0.0001. For Figure 5B in mES cells, the colocalisation of ERα-66 with GPER1 in the endoplasmic reticulum and Golgi apparatus is also significantly lower than the MCC threshold of 0.5. Hence, for M1 (endoplasmic reticulum), the value is t(46)=10.49, p<0.001 while for M1 (Golgi apparatus), the value is t(47)=10.95, p<0.001. For M2 (endoplasmic reticulum), the value is t(47)=9.012, p<0.0001 and for M2 (Golgi apparatus), the value is t(47)=10.81, p<0.0001. For M3 (endoplasmic reticulum), the value is t(47)=9.952, p<0.001 and for M3 (Golgi apparatus), t(47)=11.31, p<0.0001.

**Supplementary data 1B**: Low colocalisation in mESn of ERs is seen by statistical analyses. For Figure 6A in mESn cells, the colocalisation of ERα-66 with GPER1 in the nucleus and plasma membrane, reflected by M1, M2 and M3 values is significantly lower from the threshold of 0.5. Hence, Wilcoxon's test gives significantly lower values from the 0.5 threshold value for M1 (nucleus) at t(139)=50.27, p<0.0001 and for M1 (plasma membrane) at t(126)=29.72, p<0.001. For M2 (nucleus), the values are t(143)=43.21, p<0.0001 while for M2 (plasma membrane), the values are t(126)=29.16, p<0.0001. For M3 (nucleus), the values are t(140)=47.74 while for M3 (plasma membrane), the values are t(125)=34.76, p<0.0001.

For ERα-66 and ERα-36 (Figure 6A) in mESn cells, Wilcoxon's test reveals that colocalisation is also significantly lower from 0.5 in the nucleus and plasma membrane. Therefore, the value for M1 (nucleus) is t(141)=77.51, p<0.0001 while for M1 (plasma membrane), it is t(139)=82.50, p<0.001. For M2 (nucleus), it is t (142)=72.16, p<0.0001 and for M2 (plasma membrane), it ist(139)=83.15, p<0.0001. For M3 (nucleus), it is t(142)=77.21, p<0.0001 while for M3 (plasma membrane), it is t(137)=93.59, p<0.0001.

For Figure 6B in mES cells, colocalisaton of ERα-66 with ERα-36 in the endoplasmic reticulum and Golgi apparatus, reflected by M1, M2 and M3 values is significantly lower from the threshold of 0.5. Hence, Wilcoxon's test for M1 (endoplasmic reticulum), the value is, t(127)=8.327, p<0.0001 and M1 (Golgi apparatus), the value is t(116)=28.82, p<0.0001. For M2 (endoplasmic reticulum), the value is t(127)=7.948, p<0.0001 and for M2 (Golgi apparatus), the value is t(116)=28.85, p<0.0001. Similarly, for M3 (endoplasmic reticulum), the value is t(126)=9.576, p<0.0001 and for M3(Golgi apparatus), the value is t(115)=30.05, p<0.0001. For Figure 6B in mES cells, the colocalisation of ERα-66 with GPER1 in the endoplasmic reticulum and Golgi apparatus is also significantly lower than the MCC threshold of 0.5. Hence, for M1 (endoplasmic reticulum), the value is t(146)=12.71, p<0.001 while for M1 (Golgi apparatus), the value is t(146)=13.86, p<0.001. For M2 (endoplasmic reticulum), the value is t(146)=12.25, p<0.0001 and for M2 (Golgi apparatus), the value is t(145)=13.61, p<0.0001. For M3 (endoplasmic reticulum), the value is t(145)=12.62, p<0.001 and for M3 (Golgi apparatus), the value is t(144)=14.62 , p<0.0001.

**Supplementary data 1C**:Though there is ERα-36 and GPER1 in mES and mESn cells, there is no significant colocalisation of these two proteins in any organelle, as reflected by M1, M2 and M3 values that are significantly lower from the threshold of 0.5. Hence, Wilcoxon's test for colocalisation in mES cells (Figure 7A) gives significantly lower values from the 0.5 threshold value for M1 (nucleus) at t(49)=83.96, p<0.0001 and for M1 (plasma membrane) at t(49)=48.58, p<0.001. For M2 (nucleus), the values are t(49)=94.03, p<0.0001 while for M2 (plasma membrane), the values are t(49)=52.40, p<0.0001. For M3 (nucleus), the values are t(43)=87.99 while for M3 (plasma membrane), the values are t(49)=50.74, p<0.0001. For ERα-36 and GPER1 proteins in the endoplasmic reticulum and Golgi apparatus in mES cells (Figure 7B), Wilcoxon's test shows lower values from 0.5 threshold value for M1 (endoplasmic reticulum) at t(49)=9.861; p<0.0001 while for M2 (golgi apparatus), the values are t(49)=20.03; p<0.0001. For M2(endoplasmic reticulum), the values are t(49)=9.847 while for M2(Golgi apparatus), the values are t(49)=33.21; p<0.0001. For M3 (endoplasmic reticulum), the values are t(49)=9.948; p<0.0001 while for M3 (Golgi apparatus), the values are t(49)=18.61; p<0.0001.

Similar lack of colocalisation in seen in mESn cells. Wilcoxon's Test (Figure 7A) reveals values for M1(nucleus) to be t(46)=89.64; p<0.0001 while values for M1 (plasma membrane) are t(46)=133; p<0.0001. For M2 (nucleus), the values are t(46)=55.55; p<0.0001 while for M2 (plasma membrane), the values are t(46)=133.3; p<0.0001. For M3 (nucleus), the values are t(46)=55.95; p<0.0001 while for M3 (plasma membrane), the values are t(46)=224.1; p<0.0001.

Figure 7B shows the colocalisation of ERα-36 and GPER1 in mESn cells. Wilcoxon's test shows lower values from 0.5 threshold value for M1 (endoplasmic reticulum) at t(75)=24.97; p<0.0001 while for M2 (golgi apparatus), the values are t(101)=498.4; p<0.0001. For M2(endoplasmic reticulum), the values are t(75)=24.99 while for M2(Golgi apparatus), the values are t(101)=518.7; p<0.0001. For M3 (endoplasmic reticulum), the values are t(75)=24.99; p<0.0001 while for M3 (Golgi apparatus), the values are t(101)=405.5; p<0.0001.

These data show that in all organelles, regardless of the ERs present, no ER is colocalised with the other in that organelle.
