# Supplementary figures and images for: Localisation of oestrogen receptors in stem cells and in stem cell‐derived neurons of the mouse
Source: J Neuroendocrinol. 2022 Dec 12;35(2):e13220. doi: 10.1111/jne.13220 (PMC10909416; doi:10.1111/jne.13220)

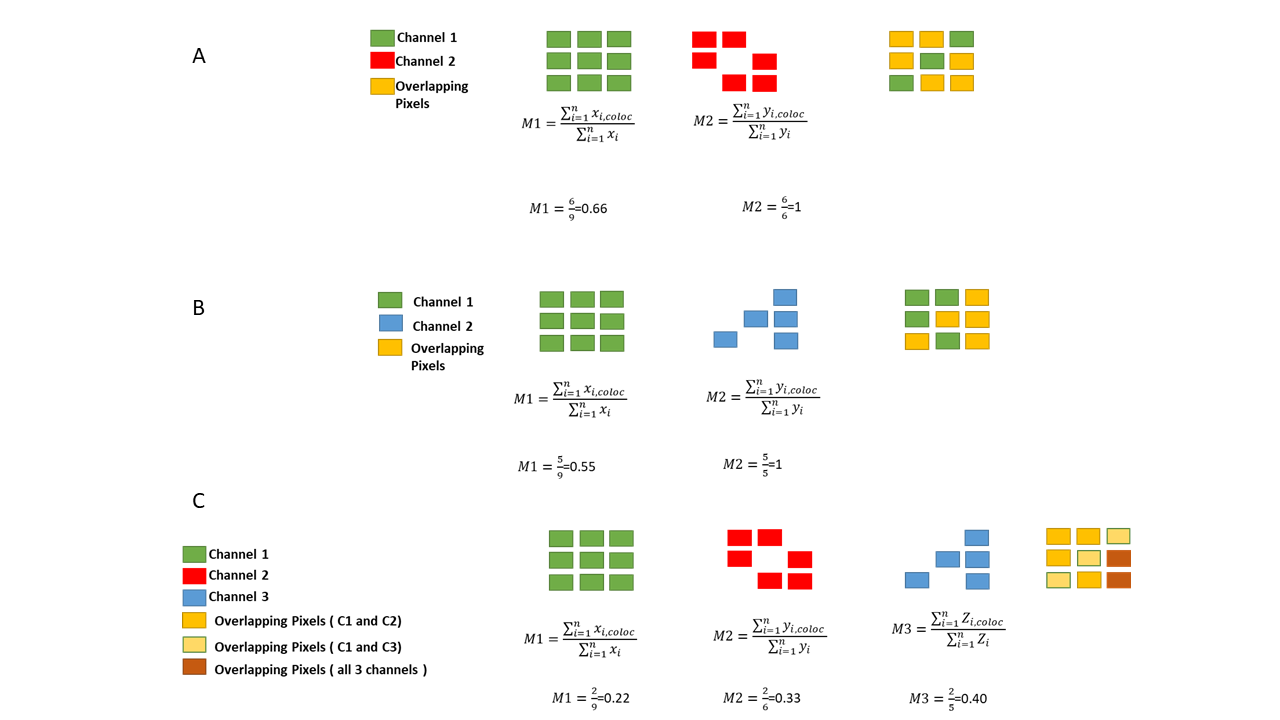

Supplement: Supplementary file 2 — FIGURE S1. An example workflow showing the intercept of different fluorophores (red, green, blue) and how colocalisation of these leads to the calculation of Manders correlation coefficients (M1, M2 or M3). For M3, the overlapping pixels for all three fluorophores is used as the numerator (C). In Figures 1, 2, 3, we sought to determine if ERs were present in different organelles. M1 always denoted the organelle stain while M2 denoted the specific oestrogen receptor antibody (ERα‐66, GPER1 and ERα‐36). Figure S1A is an example of these type of experiments If we wanted to determine if ERα was at the membrane, M1 would represent all overlapping pixels for the membrane stain and ERα as the numerator divided by the denominator that is, all pixels of the membrane stain. M2, on the other hand, would represent all overlapping pixels for the membrane stain and ERα as the numerator divided by all the pixels of ERα. If every pixel stained by the membrane stain also stained for ERα, M1 would be 1 and would represent complete colocalisation. If no pixel stained by the membrane stain overlapped with ERα staining, the numerator would be 0 and M1 would be zero. [file JNE-35-e13220-s001.tif]
